# Development of Hydrogel-Cellulose Nanofiber Hybrid Scaffolds for Potential Cutaneous Wound-Healing Applications

**DOI:** 10.3390/ijms27219650

**Published:** 2026-10-28

**Authors:** Judson V. Edwards, Nicolette T. Prevost, Haley Lassiter, Katie Hamel, Michael W. Easson, Jacobs H. Jordan, Emma Rogers, Jordan Robinson, Trivia Frazier, Cecilia G. Sanchez

**Affiliations:** 1 United States Department of Agriculture, Agricultural Research Service, Southern Regional Research Center, New Orleans, LA 70124, USA; nicolette.prevost@usda.gov (N.T.P.); micheal.easson@usda.gov (M.W.E.); jacobs.jordan@usda.gov (J.H.J.); 2 Obatala Sciences Inc., New Orleans, LA 70148, USA; haley.lassiter@obatalasciences.com (H.L.); katie.hamel@obatalasciences.com (K.H.); emma.rogers@obatalasciences.com (E.R.); jordan.robinson@obatalasciences.com (J.R.); trivia.frazier@obatalasciences.com (T.F.)

**Keywords:** cellulose nanofibrils, TEMPO-oxidized cellulose nanofibrils, human-derived extracellular matrix, hybrid hydrogel, wound healing, fibroblasts, adipose-derived stromal/stem cells

## Abstract

Cellulose nanofibrils (CNFs) and TEMPO-oxidized cellulose nanofibrils (TOCNFs) are hydrated, high-aspect-ratio biomaterials with tunable physicochemical properties, whereas human-derived extracellular matrix (ECM) hydrogels provide matrix-associated biochemical cues. In this study, CNF- and TOCNF-based hybrid scaffolds incorporating human ECM hydrogel components were developed and evaluated in vitro as platforms relevant to cutaneous wound repair. Hybrid formulations gelled at physiological temperature and exhibited formulation-dependent shear-thinning behavior, elastic properties, protein release/leaching profiles, and porous fibrillar microstructures. Selected formulations supported cell-associated metabolic activity of human dermal fibroblasts and adipose-derived stromal/stem cells under two-dimensional and three-dimensional culture conditions. Additional exploratory studies demonstrated qualitative compatibility with adipogenic culture and condition-dependent changes in wound-gap width. In the absence of in vivo validation and confirmatory biological assays, these findings are interpreted as proof-of-concept rather than evidence of wound-healing efficacy. Overall, the results demonstrate the feasibility of combining CNF or TOCNF structural networks with human-derived ECM hydrogels to generate hybrid scaffolds with wound-relevant material properties and preliminary cytocompatibility, supporting further mechanistic and preclinical evaluation.

## 1. Introduction

Cutaneous wound repair requires coordinated interactions among extracellular matrix (ECM), resident and infiltrating cells, soluble mediators, vascular elements, immune responses, and, in open wounds, microbial communities. Biomaterial scaffolds cannot recreate this complete microenvironment. Historically, three-dimensional scaffolds have instead been used to mimic selected aspects of native ECM and connective-tissue architecture, thereby providing structural and biophysical cues relevant to cellular behavior [1,2,3,4,5].

Cellulose nanofibrils (CNFs) and TEMPO-oxidized cellulose nanofibrils (TOCNFs) are high-aspect-ratio cellulose structures with nanoscale diameters and lengths that can extend into the micrometer range [6,7,8,9,10,11,12,13]. Their hydrated networks, surface chemistry, porosity, and shear-dependent flow behavior make them attractive for moist wound interfaces and cell-supporting scaffolds. Nanocellulose and related cellulose-based biomaterials have been reviewed for wound-dressing applications [14,15,16], and nanofibrillar cellulose dressings have been evaluated in skin-repair settings [17]. Other cellulose-based platforms evaluated in wound or tissue-engineering contexts include cellulose acetate composites [18,19], bacterial cellulose [20,21], cellulose nanocrystal-containing hydrogels [22,23], and cellulose-based composite scaffolds [24]. Related studies have characterized the microstructure, mass transport, and wet-state mechanics of nanocellulose materials [25,26,27], while cellulose filament architecture provides additional context for geometry-dependent material behavior [28]. Clinical evaluation of a nanofibrillar cellulose dressing and in vitro studies of cellulose-containing polysaccharide nanofibers further support the relevance of cellulose platforms to wound interfaces and fibroblast responses [29,30].

CNF and TOCNF are biocompatible structural materials, but cellulose alone does not reproduce the compositional complexity of human ECM. Decellularized matrices provide a general tissue-engineering framework [31], XGel and adipose-derived ECM materials have been characterized for stem-cell culture, differentiation, composition, microstructure, and remodeling [32,33,34,35]. Human adipose-derived cells have also been evaluated in two- and three-dimensional in vitro fat constructs [36]. A broader review has considered adipose-derived, blood-derived, and cellulose-based materials in skin regeneration [37]. These findings support combining nanocellulose-mediated structure and mechanics with the biochemical composition of human-derived ECM in a single hybrid scaffold.

The scientific gap is the limited comparative information on how unmodified CNF and TOCNF behave when separately combined with human-derived ECM across formulation ratios, and how the resulting hybrid materials relate to rheology, microstructure, cell-associated metabolic activity, adipose-derived stem cell compatibility, and an exploratory in vitro wound-gap response. The purpose of this study was to formulate and characterize CNF-human ECM and TOCNF-human ECM hybrid scaffolds, identify a lead formulation using a combined physicochemical and handling framework, and define the scope and limitations of their in vitro biological performance. The study does not evaluate clinical efficacy, direct structural degradation, antimicrobial activity, inflammatory modulation, or in vivo wound repair.

## 2. Results

### 2.1. Hydrogel Composition, Visual Characteristics, Protein Release, and Proteomics

Cellulose nanofibrils (CNFs) and TEMPO-oxidized cellulose nanofibrils (TOCNFs) derived from wood and cotton were used and had been characterized previously [38,39]. Consistent with accepted nanocellulose nomenclature, CNFs may possess nanoscale diameters while extending into the micrometer range, resulting in high-aspect-ratio structures. Supplementary Figure S1 shows cotton-derived fibrils with micrometer-scale lengths of approximately 1–2 µm. This dimension represents fibril length.

**Figure 1:**
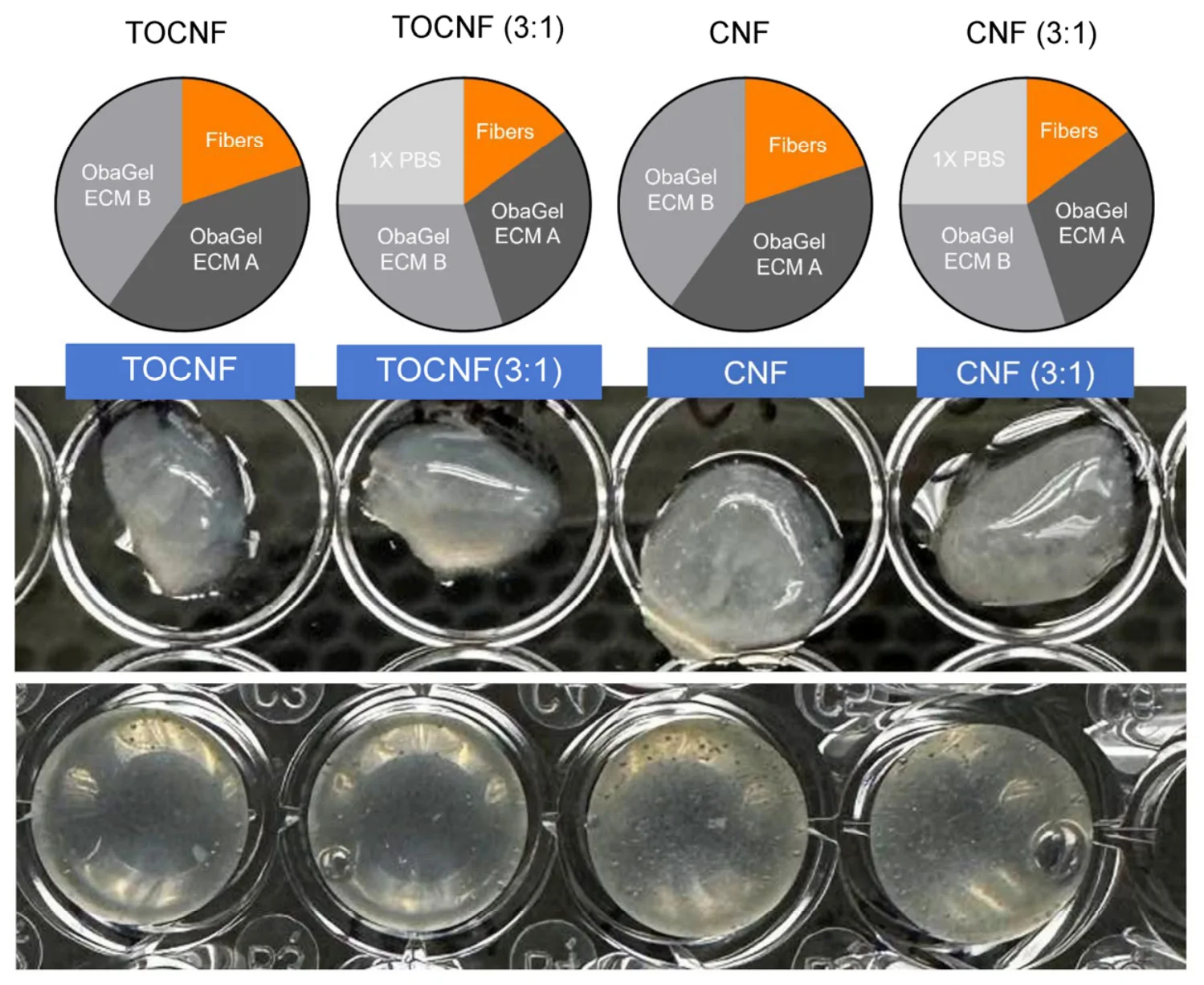
Visual presentation of formulation #4 hydrogels for TOCNF, TOCNF 3:1 dilution, CNF, and CNF 3:1 dilution after 30 min at 37 °C.

Comparative screening of formulations #1–#4 informed the selection of the lead composition (Figure 1). Formulations containing two types of cellulose nanofibers (CNF or TOCNF) and ECM mimetics were prepared at volume mixing ratios shown in Table 1, corresponding to nanofiber weight percentages of 2–5%, based on a 10% nanofiber stock solution. Thus, CNF and TOCNF scaffolds were separately combined with blood-derived ECM using four formulation ratios. In terms of physical appearance, CNF and TOCNF were homogeneous in solution, with no precipitation observed (Figure 1). The homogeneous distribution of TOCNF in ECM formulations 1–4 improved hydrogel structural stability and provided facile anchoring points for cell adhesion. TOCNF was the easiest to visualize because it was the most transparent. Figure 1 presents mixture #4 for TOCNF and CNF, along with their diluted counterparts, TOCNF (3:1) and CNF (3:1), to support transparency and gelation.

**Figure 2:**
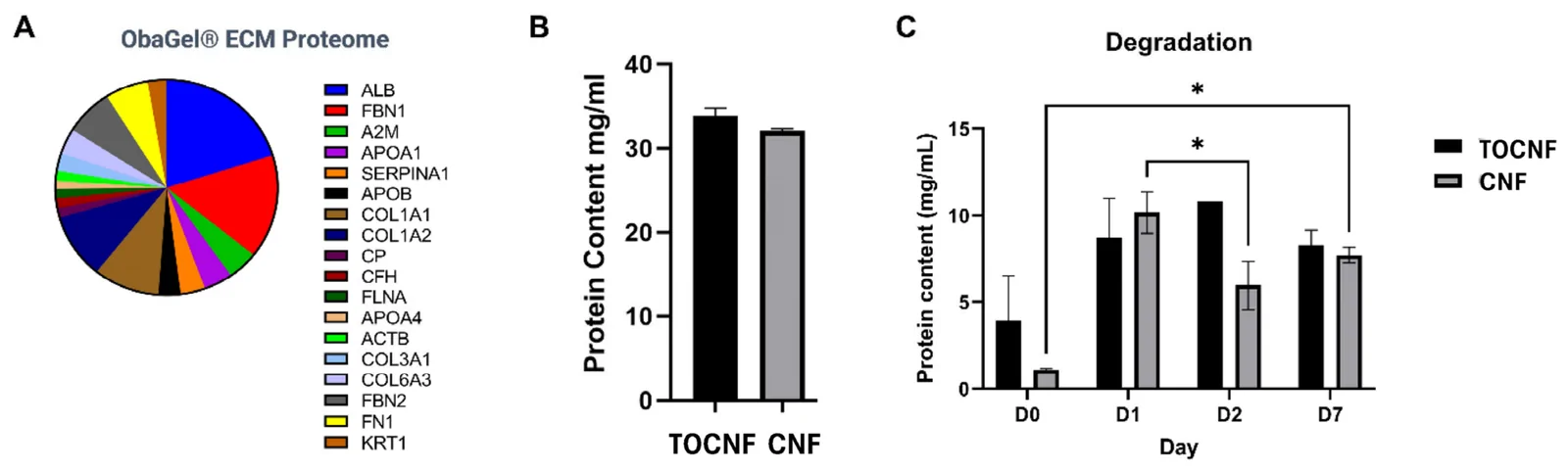
(**A**) ObaGel^®^-ECM proteomic profile showing an extracellular matrix protein-dominant composition. (**B**) Protein content of CNF and TOCNF formulation #4 pre-gelation mixtures measured using the Pierce BCA protein assay, reported as mean ± standard deviation from 3 replicates. (**C**) Protein release/leaching from CNF and TOCNF formulation #4 constructs assessed in PBS supernatants on days 0, 1, 2, and 7 using the BCA assay. Statistical testing was performed using two-way ANOVA GraphPad Software, Boston, MA, USA) with Tukey multiple-comparison testing. These measurements are interpreted as protein release/leaching and not as direct evidence of bulk scaffold degradation. Statistical significance as: * *p* < 0.05.

Proteomic analysis of the ECM component identified ECM-associated proteins, cytokines, and growth factors, including collagen and fibronectin (Figure 2A). We confirmed the nature and composition of the ECM component, as previously reported [37]. Proteomic analysis was also performed on the nanofibers. Due to processing during collection and manufacture of the nanofibers, multiple human proteins were detected in the analysis. Nevertheless, one common protein was identified in both CNF and TOCNF: a 53-kDa protein from *Gossypium hirsutum*, ribulose bisphosphate carboxylase large chain P14958. Total protein content for both nanofibril formulation #4 groups was similar, as shown in Figure 2B. Finally, total protein detected in PBS supernatants over time was interpreted as protein release or leaching and not as a direct measure of bulk structural degradation, as shown in Figure 2C.

### 2.2. Rheological Characterization

**Figure 3:**
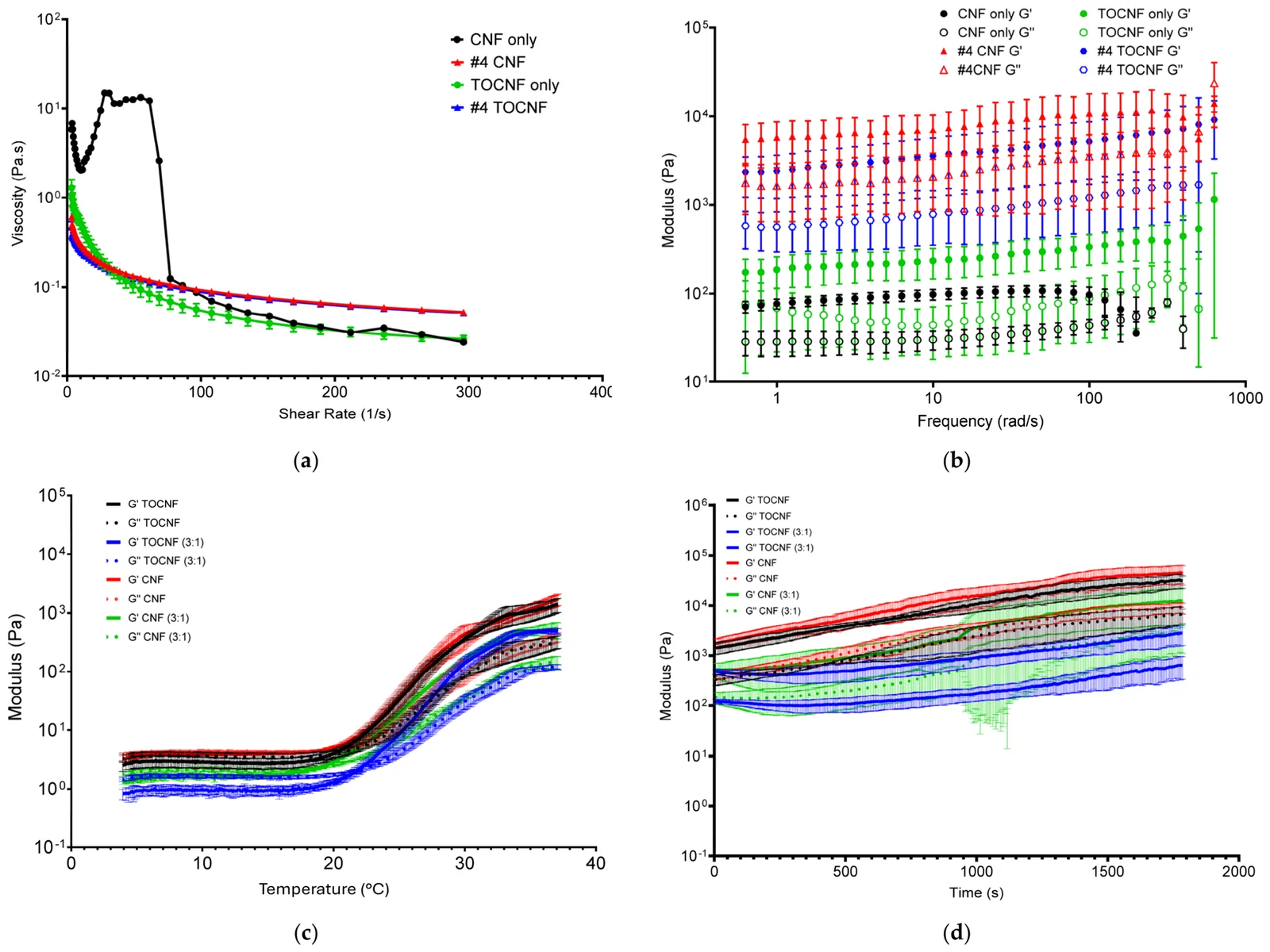
Rheological characterization of CNF and TOCNF hydrogel conditions. (**a**) Viscosity of pre-gelation hydrogel mixtures measured by flow ramping from 1 to 100 s^−1^ at 4 °C, the handling temperature used to limit premature gelation. (**b**) Storage modulus (G′, solid symbols) and loss modulus (G″, open symbols) of crosslinked hydrogels measured by frequency sweep from 0.1 to 100 Hz at 1% strain. (**c**) Storage and loss moduli for formulation #4 and 3:1 diluted formulation #4 during temperature ramping from 4 to 37 °C at 1 °C/min. (**d**) Storage and loss moduli during the 1800 s post-soak step at 37 °C, 1 Hz, and 1% strain. Measurements were performed with 3 replicates.

Figure 3 summarizes the rheological characterization of CNF, TOCNF, formulation #4, and 3:1 diluted formulation #4 conditions, including viscosity, frequency-sweep, temperature-ramp, and post-soak measurements.

The viscosity profiles of CNF and TOCNF alone and formulation #4 of CNF and TOCNF are shown in Figure 3a. CNF, TOCNF, and formulation #4 mixtures displayed shear-dependent flow behavior. Notably, the 1% (*w*/*v*) CNF-only solution showed a sharp decrease, most likely attributable to aggregation of the CNF-based material. This observation is consistent with the tendency of carboxylate-free CNF to form aggregates and, in the presence of cells, to form only weak cellular adhesions on the surface [17]. In contrast, this behavior was not observed with 1% (*w*/*v*) TOCNF alone.

The #4 TOCNF formulation produced smooth asymptotic viscosity/shear curves, with sharp increases in viscosity at low shear rates followed by a gradual decrease at high shear rates. Storage and loss moduli were measured across frequency, and temperature-ramp testing was used to evaluate sol-gel behavior. As shown in Figure 3a,b, formulation stiffness changed after 20 °C and then stabilized at 37 °C following a temperature ramp of 1 °C/min over a 10-min period. The elastic shear modulus (Gʹ, storage modulus), shown in Table 2, was within a range comparable to previous reports for CNF, TOCNF, and other biopolymers, such as alginate [40]. Notably, the stiffness of the hydrogel formulations was also similar to previously reported elastic shear modulus values for mesenchymal stem cells (MSCs) cultured on CNF films [6].

TOCNF formulation #4 showed lower storage and loss moduli than the higher-nanofiber TOCNF formulations #2 and #3, as shown in Supplementary Table S1. This selection identifies the lead condition within the tested design space and does not establish universal optimality. The data support formulation-dependent, shear-dependent and elastic behavior within the tested conditions.

Rheological measurements are summarized in Table 2.

### 2.3. SEM Image Analysis

**Figure 4:**
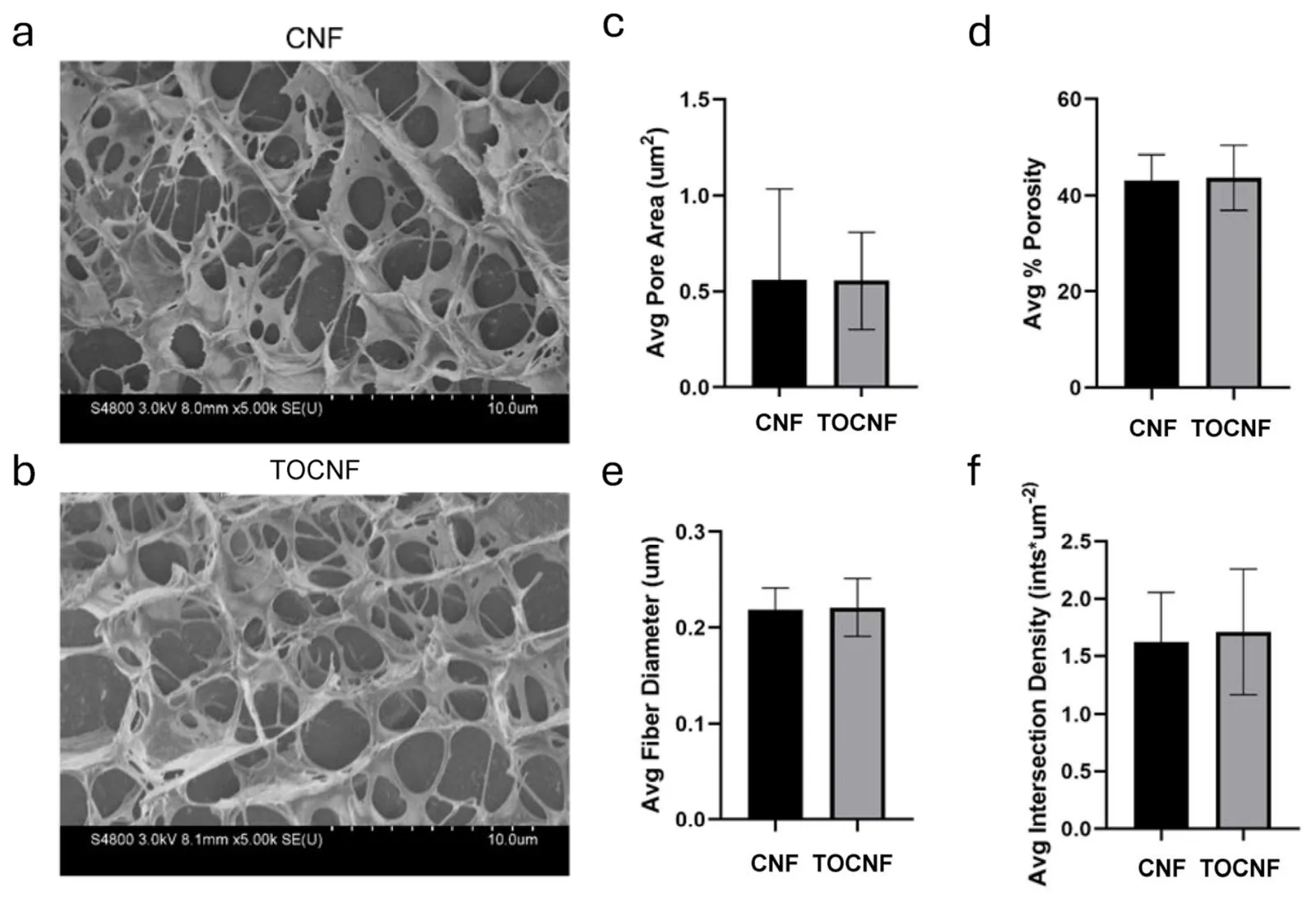
(**a**,**b**) Representative SEM images of CNF and TOCNF formulation #4 hydrogels showing porous fibrillar microstructures. Hydrogels were cross-linked overnight in 48-well plates at 37 °C and 5% CO2 and imaged using a Hitachi S-4800 SEM equipped with a Gatan Alto 2500 cryogenic SEM system, as described in Section 4. Scale bar = 10 µm. Pore area (**c**), percent porosity (**d**), fiber diameter (**e**), and intersection density (**f**) were estimated using the plugin DiameterJ ImageJ/FIJI plugin, NIH ImageJ Team, Bethesda, MD, USA. Two-way ANOVA with Tukey multiple-comparison testing did not identify statistically significant differences among the hydrogel conditions; therefore, these images are interpreted descriptively.

Representative SEM images of CNF and TOCNF formulation #4 showed porous fibrillar microstructures (Figure 4a). ImageJ/DiameterJ analysis of pore area, percent porosity, fiber diameter, and intersection density revealed no significant differences among hydrogel conditions by two-way ANOVA (GraphPad Software, Boston, MA, USA) with Tukey multiple-comparison testing (Figure 4c–f); visible variation is therefore descriptive rather than evidence of structural differences.

### 2.4. Cell-Associated Metabolic Activity

**Figure 5:**
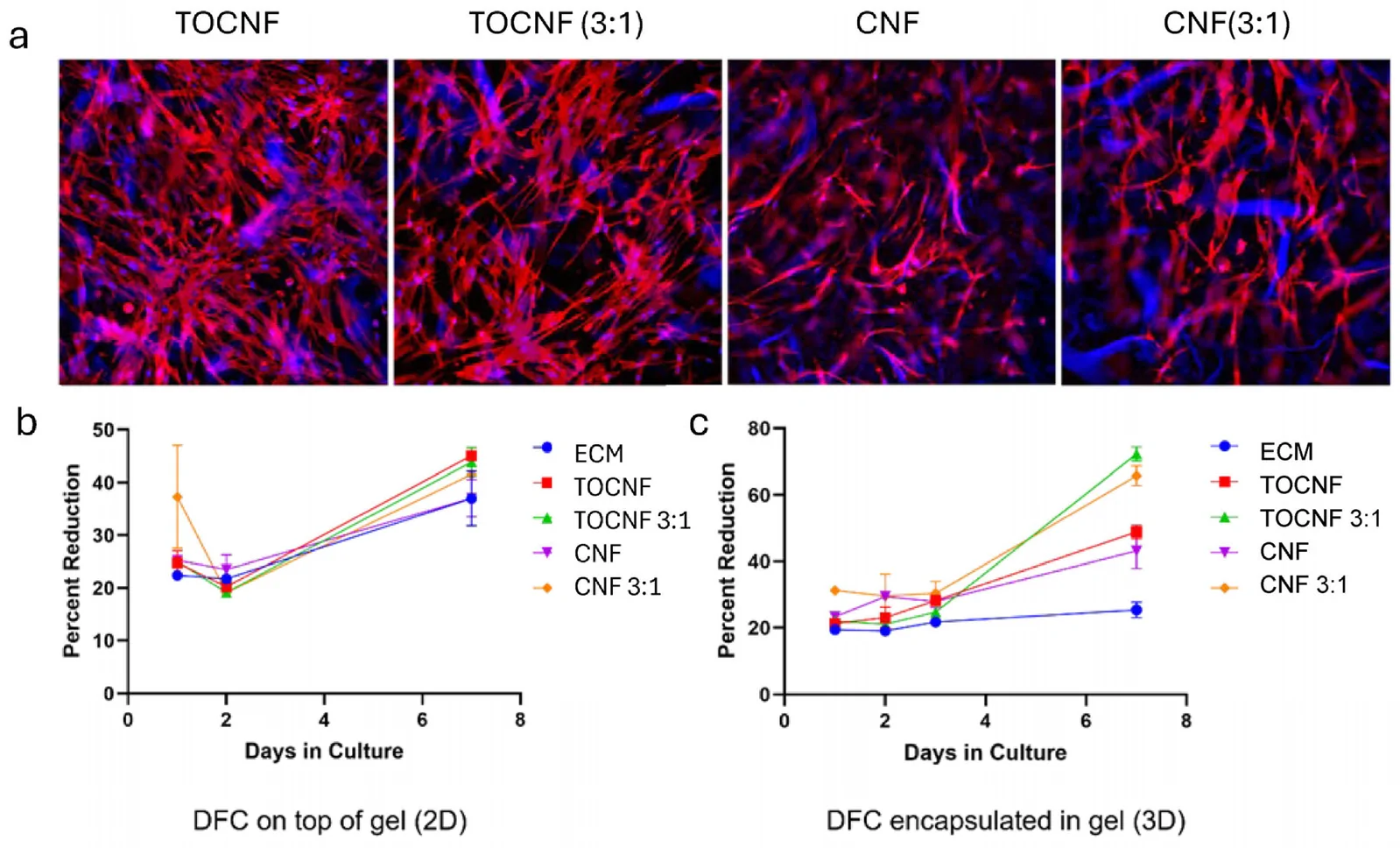
Dermal fibroblast cells (DFC)s cultured on the surface or encapsulated in the CNF and TOCNF # 4 mix hydrogels. (**a**) Representative images of DFC after 7 days of culture on hydrogel coated well plates and stained with Alexa Fluor Phalloidin 594 (red = actin) and NucBlue ReadyProbes (Hoechst 33342) (blue = nuclei). (**b**,**c**) Cell-associated metabolic activity of human dermal fibroblasts cultured in two and three-dimensional hydrogel formulations. Two biologically independent studies were conducted, each with three technical replicate wells per condition on days 1,3 and 7 (2D studies) and days 1, 2, 3, and 7 (3D studies). Acellular scaffold controls were used for background correction and were not plotted separately.

As shown in Figure 5, human dermal fibroblasts cultured on the surface of or encapsulated within formulation #4 hydrogels showed cell-associated metabolic activity over days 1–7, assessed by alamarBlue reduction after correction using acellular controls. These measurements are not direct cell counts or evidence of proliferation. Complementary nuclear staining quantification yielded 133.7 ± 48.1 nuclei/field for CNF and 166.3 ± 6.0 nuclei/field for TOCNF, with coefficients of variation of 36.0% and 3.6%, respectively (Supplementary Table S2). Experimental replication and image-sampling procedures are described in Section 4.7 and Section 4.8. Together, these findings support compatibility with human dermal fibroblasts.

### 2.5. Adipogenic Differentiation

**Figure 6:**
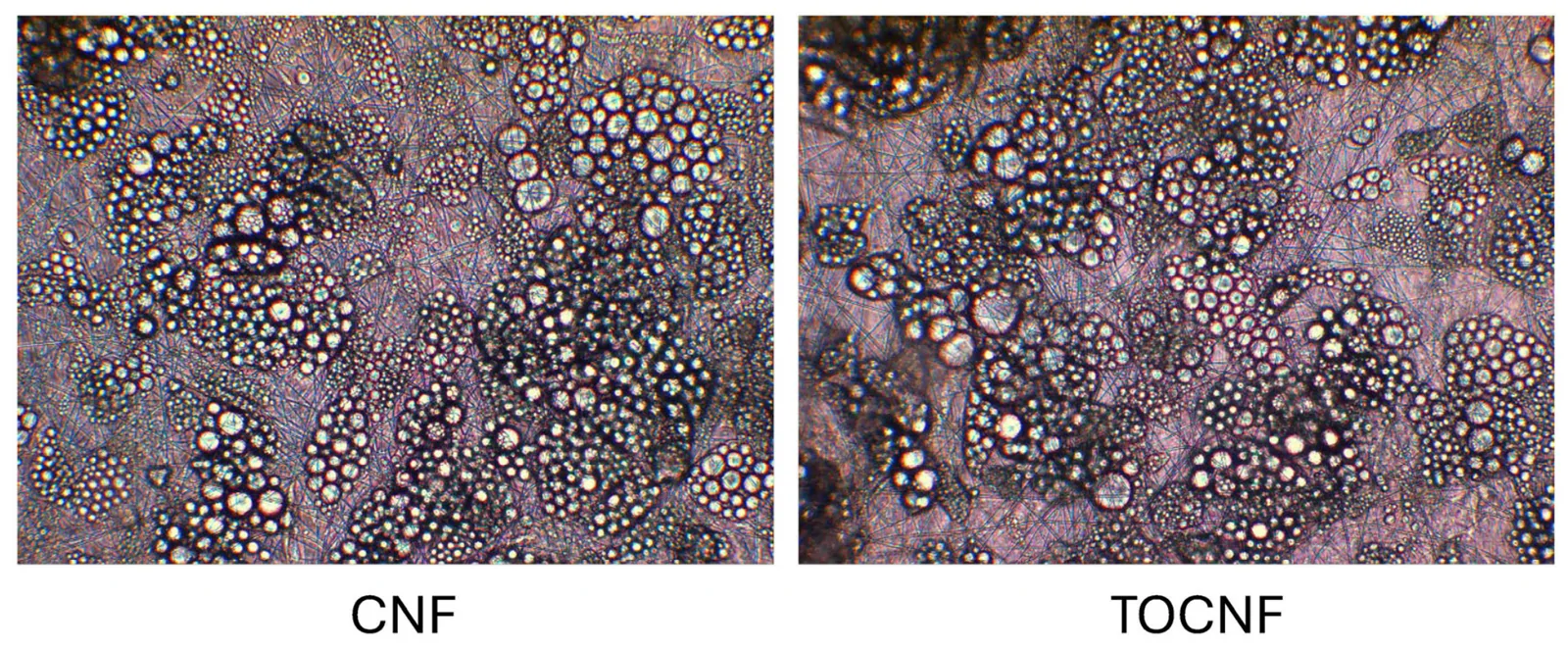
Representative brightfield images from a qualitative adipogenic-compatibility study. Label-free intracellular lipid droplets were detected after 31 days in adipogenic medium. No quantitative comparison between CNF and TOCNF was established.

In a 31-day feasibility study, ASCs cultured on PCL NanoECM^®^ fibers coated with formulation #4 CNF or TOCNF were maintained for 7 days and then induced into adipogenesis in Adipoqual media. Preadipocytes showed label free intracellular lipid droplets under adipogenic conditions and robust adipogenesis was observed at day 21, maintained at day 31 (Figure 6). These qualitative observations support long-term adipogenic compatibility but do not establish differences in lipid-droplet size, extent of adipogenesis, or formulation superiority.

### 2.6. Exploratory Wound-Gap Closure Assay

**Figure 7:**
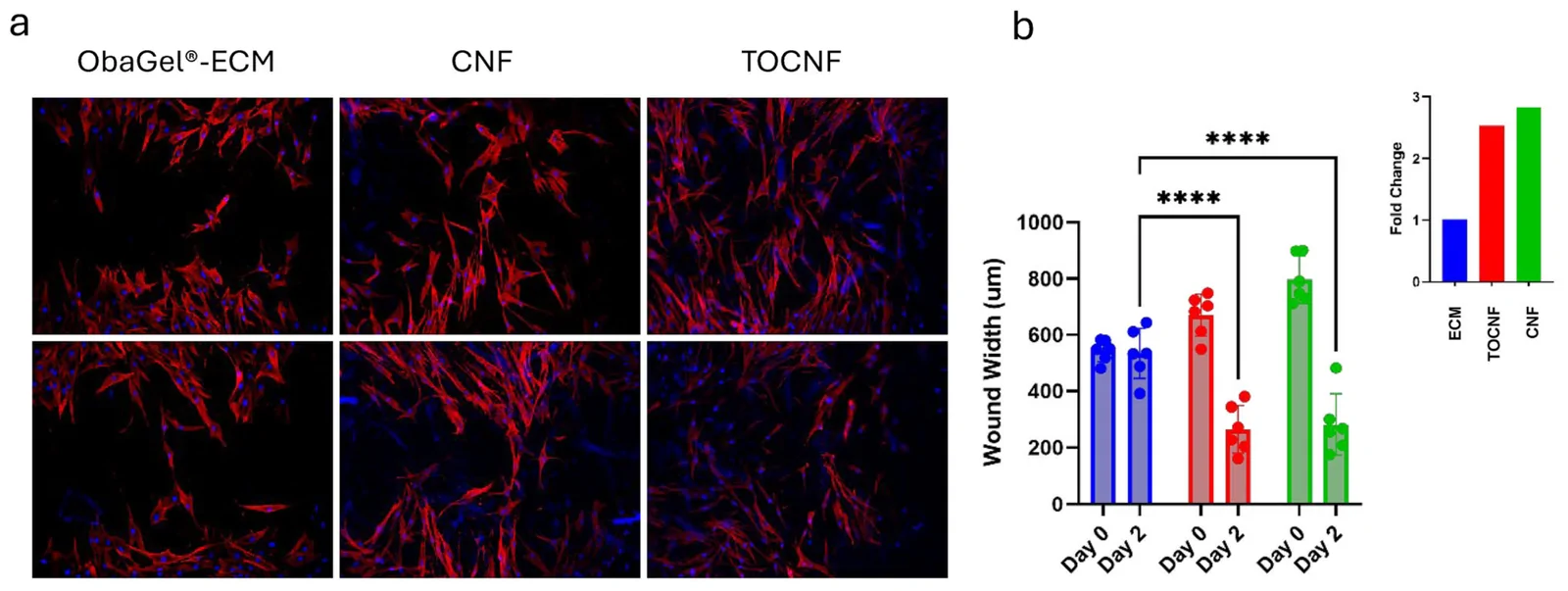
Exploratory wound-gap closure assay. (**a**). Representative images for gap closure. (**b**). Wound-width measurements reflect combined cellular processes. Data represents independent biological replicates with triplicates per assay. Statistical significance as: **** *p* < 0.0001.

A exploratory wound gap analysis is presented in Figure 7, using ASCs as cellular model. Changes in wound width reflect the combined effects of cell movement, spreading, survival, and proliferation and are therefore a composite wound-gap response rather than a migration-specific endpoint. Individual wound-width values on days 0 and 2 and the corresponding fold changes are presented for ObaGel^®^-ECM, formulation #4 CNF, and formulation #4 TOCNF conditions. On day 2, wound closure was 1.9 ± 2.4% for ObaGel^®^-ECM, 64.3 ± 5.4% for formulation #4 CNF, and 60.0 ± 9.5% for formulation #4 TOCNF (Supplementary Figure S2 and Table S3). Representative Hoechst/phalloidin images show a persistent open region in the ObaGel^®^-ECM condition and greater cellular occupation of the wound region in the CNF and TOCNF conditions (Figure 7).

## 3. Discussion

### 3.1. Scope of the Findings

This study demonstrates that CNF and TOCNF can be formulated separately with human-derived ECM components to produce hybrid hydrogels with measurable rheological properties, porous morphology, and compatibility with selected human cell types in vitro. The findings apply to the tested formulations and assays; translational limitations are detailed in Section 3.6.

### 3.2. Hybrid Scaffold Rationale and Formulation Selection

Modified and surface-carboxylated cellulose materials have been investigated as cell-culture scaffolds, and cellulose geometry can influence composite mechanical behavior [41,42,43]. In consequence, cellulose materials have been studied as tools for biomolecule and enzyme immobilization, although this function was not evaluated here [44], antibacterial applications [45,46,47], wound materials [14,17], and macrophage responses to surface-modified nanofibrillar cellulose [48] providing relevant context.

The hybrid design described in this study, combines the hydrated fibrillar structure and formulation-dependent mechanics of CNF or TOCNF with the biochemical complexity of human-derived ECM. In this study formulations containing CNF or TOCNF were screened using gelation, nanofiber loading, rheology, transparency, handling, and preliminary cell compatibility. The observed cell-associated responses in this study support the biocompatibility of the formulations and potential use as biomaterial for wound healing applications. Protein detected in the supernatant indicates release or leaching from the constructs. Nevertheless, it does not, by itself, establish polymer-network degradation, mass loss, erosion kinetics, or retention of mechanical integrity.

### 3.3. Cell-Associated Activity and Wound-Gap Closure

Dermal fibroblasts were cultured either on the surface of or encapsulated within nanocellulose-containing hydrogel mixture and their metabolic activity was measured over several days. The alamarBlue readout provides a measure of reduction associated with metabolically active cells but is not necessarily equivalent to cell numbers or proliferation. The complementary DAPI analysis in Supplementary Table S2 provides independent nuclear-density measurements, supporting the biocompatibility studies, while Supplementary Figure S2 and Table S3 provide quantitative and qualitative support for wound-gap observations. In the wound gap model, reduced wound gap was observed on both TOCNF and CNF #4 formulations compared to ObaGel^®^ ECM alone. No cellular toxicity was observed. Those results confirm previous studies on TOCNF. TOCNF has shown no toxicity toward mouse embryonic fibroblast cell lines derived from Swiss albino or BALB/c mice, or toward primary human skin cells, including dermal fibroblasts and keratinocytes [30]. CNFs also adhere to the wound bed and support re-epithelialization [17,18,20,29].

These mechanical and biological properties could support wound healing applications by improving fluid uptake and retention, maintaining hydration, preserving flexibility, and enabling tunable dressing porosity. When used with extracellular matrix (ECM) components, nanocellulose may therefore act synergistically to promote wound healing at the biophysical level. This interpretation is consistent with CNF-based dressing designs in which dressing architecture can be tuned [15,16,17,18,19], and it suggests further potential for ECM-incorporated formulations.

### 3.4. Mechanics and Adipogenic Compatibility

Matrix stiffness, viscoelasticity, and related physicochemical properties can influence stromal-cell behavior and fate [6,8,40,49,50]. The present study characterized formulation-dependent rheology but did not vary mechanical properties independently of composition; therefore, it does not establish a causal relationship with ASC growth or differentiation. The qualitative lipid accumulation observed with formulation #4 is consistent with reports linking soft matrices to adipogenic differentiation. In hydrogel gradients, mesenchymal stem-cell adipogenesis has been reported at approximately 1.9 kPa [6,8], a compliance range relevant to native adipose tissue; related studies associate soft matrices with adipogenic-marker expression and lipid-bearing adipocytes [50]. Prior XGel and ObaGel studies provide additional context [32,36].

### 3.5. Study Limitations and Next Steps

The limitations include lot-to-lot variability in multiple biological replicates; orthogonal proliferation measurements; quantitative assessment of lipid droplets/cells; absence of antimicrobial, biofilm, inflammatory, and immune-cell assays; and absence of in vivo wound-healing studies. No human clinical testing was performed. Human cells and source tissues were used only in in vitro assays and for preparation of human-derived materials. Future studies should combine release measurements with gravimetric analysis, swelling, rheology over time, structural imaging, and biochemical integrity assays. Translation will require reproducible manufacturing quality control, donor- and lot-level characterization, adequately powered multi-donor studies, animal or alternative validated preclinical wound models as appropriate, and direct assessment of infection and inflammation.

### 3.6. Wound-Healing Relevance and Translational Context

The hybrid scaffold design builds on characterization of XGel, a component of ObaGel ECM, for stem-cell expansion and tissue engineering [32], adipose-derived matrices for composition, microstructure, and remodeling [33,34,35,36], and human adipose-derived cells in two- and three-dimensional fat constructs [36]. These platforms support the design rationale. In addition, evaluation of decellularized adipose tissue hydrogel in a murine bone-regeneration model provides broader regenerative-medicine context [51].

Prior studies of adipose-derived stromal/stem cells, stromal vascular fraction, processed microvascular tissue, and human-derived matrices provide a biological rationale for wound-repair applications [33,34,35,37,52,53,54,55]. In murine pressure-ulcer models, adipose stromal cells accelerated closure, improved epidermal and dermal architecture, increased adipogenesis, and reduced inflammatory-cell infiltration [55]. Related studies evaluated the safety of human SVF and the safety and efficacy of human ASCs [52,53], while processed microvascular tissue improved closure and was associated with inflammatory and vascular remodeling responses [54]. These findings support the wound-healing relevance of adipose-derived biological materials.

Future preclinical evaluation should quantify wound histology, tissue architecture, angiogenesis, inflammatory responses, matrix remodeling, and scaffold persistence. The standardized murine-wound-histology scoring system provides one framework for this assessment [56], which is needed to determine whether the observed material properties and in vitro compatibility translate into regenerative benefit.

## 4. Materials and Methods

### 4.1. Materials and ECM Source

ObaGel^®^-ECM A (cat. no. OS-301) and ObaGel^®^-ECM B (cat. no. OS-314) are proprietary human blood-derived hydrogel ECM materials manufactured by Obatala Sciences Inc., New Orleans, LA, USA and are related to the previously characterized Obagel^®^ and XGel human-derived hydrogels [32,36]. Source tissues were obtained from consented donors through the National Disease Research Interchange under protocols RGIJ1 01 001A and 002A. The present study did not perform a formal lot-to-lot variability analysis. Unless otherwise indicated, experiments were performed in triplicate. TOCNF and CNF were obtained from the University of Maine Process Development Center as freeze-dried powders. Cotton CNF was produced from cotton gin mote cellulose as previously described [39].

### 4.2. Fabrication of Hydrogel Blends

CNF and TOCNF stock suspensions were prepared at 1% (*w*/*v*; 10 mg/mL) in cell-culture-grade deionized water and equilibrated to pH 7.5 with 10× PBS. Each nanofibril type was separately blended with ObaGel ^®^ ECM A and ECM B at 5:1:4, 3:1:6, 24:1:25, and 1:2:2 ratios. Nanofibril-only suspensions were included as gelation controls. Aliquots of 500 µL were incubated for 30 min at 37 °C, and gelation was recorded visually as yes or no.

### 4.3. Rheological Analysis

Rheological measurements were performed using a Discovery TA Discovery HR-2 rheometer (TA Instruments, New Castle, DE, USA) with a 25 mm parallel plate, 500 µm gap, and 350 µL sample volume. Viscosity was measured from 1 to 100 s^−1^ at 4 °C. Frequency sweeps were performed from 0.1 to 100 Hz at 1% strain. Temperature-ramp testing increased the sample temperature from 4 to 37 °C at 1 °C/min, followed by an 1800 s hold at 37 °C at 1 Hz and 1% strain. Rheological measurements used *n* = 3 replicates.

### 4.4. Hydrogel Protein Content

Hydrogel protein content was measured using the Pierce BCA Protein Assay Kit (Cat # 23225; Thermo Scientific, Waltham, MA, USA). Selected hydrogel formulations were aliquoted into microtubes and diluted 1:10 in radioimmunoprecipitation assay lysis and extraction buffer (Cat #: R0278-50mL; Sigma Aldrich, Saint Louis, MO, USA). Bovine serum albumin standards and reagents were prepared according to the manufacturer’s protocol, absorbance was measured at 560 nm using a BioTek Synergy H1 microplate reader (Agilent, Santa Clara, CA, USA).

### 4.5. Protein Release Study

Each hydrogel formulation (250 µL) was gelled in a 48-well plate for 15 min at 37 °C and 5% CO_2_. PBS (250 µL) was added to each construct. The plate was incubated at 37 °C in a carbon dioxide (CO_2_) incubator (ThermoForma, Marietta, OH, USA, Model #3110) set at 5% CO_2_ for 15 min to initiate gelation. Once the constructs gelled, 250 µL of 1× PBS was added to the surface of each construct. On days 0, 1, 2, and 7, 50 µL of supernatant was collected and replaced with fresh PBS. Collected samples were stored at −20 °C and analyzed using the BCA protein assay kit (Thermo Fisher, Waltham, MA, USA, cat. no. 23225) to quantify protein released into the PBS solution. This method quantified released protein and was not used as a direct measure of structural degradation.

### 4.6. Scanning Electron Microscopy

Hydrogel aliquots (150 µL) were cross-linked in 48-well plates overnight at 37 °C and 5% CO_2_. Samples were transported at room temperature to the Tulane University Coordinated Instrument Facility and imaged using a Hitachi S-4800 SEM equipped with a Gatan Alto 2500 cryogenic SEM system (Gatan, Pleasanton, CA, USA), following the previously reported cryogenic imaging procedure [32]. The available study record does not provide additional dehydration, coating, or fracture steps. Images were segmented using DiameterJ, an open-source ImageJ/FIJI plugin, NIH ImageJ Team, Bethesda, MD, USA. for automated analysis from scanning electron microscope (SEM) images to estimate fiber diameter, intersection density, pore area, and percent porosity [57].

### 4.7. Dermal Fibroblast Culture on Hydrogel Coatings

Hydrogel-coated wells were prepared using ECM, formulation #4 CNF, formulation #4 TOCNF, and their 3:1 dilutions. Human dermal fibroblasts were seeded at 10,000 cells/well. AlamarBlue assay was performed using a 1:10 dilution of alamarBlue (cat. no. A50100; Invitrogen, Waltham, MA, USA) in StromaQual™ medium and incubated at 37 °C following manufacturer’s instructions. Two independent biological experiments were performed. In each experiment, at least three individual alamarBlue measurements at each time point were collected and analyzed in triplicates (wells) with three reading per well.

AlamarBlue reduction was measured on days 1, 2, and 7, after 4 h incubation with the reagent. After incubation, 100 µL of each reduced sample was transferred to a clear-bottom, black, 96-well plate. Absorbance and fluorescence were measured at 570/600 nm and 560/590 nm (ex/em), respectively, using the Biotek Synergy H1 microplate reader (Agilent, Santa Clara, CA, USA). The remaining culture medium was removed from the wells, and DFCs were fixed with 10% neutral buffered formalin, washed with 1× PBS, stained with Hoechst 33342 (Thermo Fisher, cat. no. R37605) and imaged using a Motic AE2000 phase-contrast microscope (Motic, Schertz, TX, USA) and analyzed using Fiji (ImageJ) 1.53j, NIH ImageJ Team, Bethesda, MD, USA.

Nuclear density was assessed across three independent biological triplicates per condition; three microscopic fields were analyzed per well and averaged within each biological replicate. Results were summarized as mean nuclei per field, standard deviation, standard error of the mean, and coefficient of variation (Supplementary Table S2). The coating assay was interpreted as an exploratory assessment of cell-associated metabolic activity, cellular density, and morphology rather than as a direct measurement of proliferation.

### 4.8. Dermal Fibroblast Culture in 3D

Human dermal fibroblasts were mixed with each hydrogel formulation and transferred to 96-well plates at 10,000 cells/well. alamarBlue assay was measured after a 24 h incubation with the reagent in independent wells on days 1, 2, 3, and 7. Two biologically independent studies were conducted separately from manufacturing to analysis. Within each study, every condition was evaluated in three technical replicate wells at each of the four time points, with triplicate instrumental readings per well. Triplicate instrumental readings were averaged for each well and were not treated as independent biological replicates. Acellular/no-cell scaffold controls were included at each time point for background correction, not plotted as separate experimental groups. The alamarBlue readout was interpreted as cell-associated metabolic activity rather than as a direct measure of cell number or proliferation.

### 4.9. Stem-Cell Proliferation and Differentiation

Human adipose-derived stromal/stem cells (ASC)s (Obatala Sciences, cat# OS-101) were seeded at 89,000 cells/well of a polycaprolactone (PCL) NanoECM^®^ 24-well plate (Neuromics, cat# 2401, Edina, MN, USA) coated with hydrogel mixtures #4 CNF or TOCNF described in Table 1. ASCs were maintained in StromaQual™ (Obatala Sciences, cat. no. OS-001) for 11 days followed by adipogenic differentiation with AdipoQual™ (Obatala Sciences, cat. no. OS-002) for 31 days.

### 4.10. Exploratory Predicted-Gap-Closure Assay

Human ASCs were seeded at 100,000 cells/well on 12-well plates coated with ObaGel^®^-ECM B, formulation #4 CNF, or formulation #4 TOCNF. Inserts from the Abcam Wound Healing Assay kit (cat. no. ab242285) were used to establish a defined cell-free gap. Cultures were maintained at 37 °C, 5% CO_2_ until confluency was reached. After confluency, cells were fixed with 4% formalin, and the cytoskeleton and nuclei were stained with Alexa Fluor Phalloidin 594 (Thermo Fisher, cat. no. A12381) and Hoechst 33342 (Thermo Fisher, cat. no. R3760), respectively, according to Obatala Sciences’ standard operating procedure. Inserts were removed to initiate gap study, and images were evaluated on Day 0 and Day 2. Day 0 and Day 2 measurements were paired within the corresponding condition and biological replicate. Wound closure was calculated as [(W0 − W2)/W0] × 100, where W0 and W2 represent the Day 0 and Day 2 wound widths, respectively. At the Day 2 endpoint, cultures were fixed with 4% formalin (Cat # 2 531201; Richard-Allan Scientific, Kalamazoo, MO) for 30 min at RT. Cells were stained with Alexa Fluor Phalloidin 594 and Hoechst 33342. Wound width was measured in Fiji (ImageJ) 1.53j, NIH ImageJ Team, Bethesda, MD, USA at three representative regions per image, and outcomes were summarized as mean ± SD (Supplementary Figure S2B). Phalloidin-positive area was measured within a standardized central predicted-gap region corresponding to the middle 50% of each endpoint field using identical red-channel intensity and channel-dominance criteria across conditions (Supplementary Figure S2C).

### 4.11. Human-Derived Materials and Ethical Statement

No human participants were enrolled, and no human clinical testing was performed. Source tissues used to generate human-derived materials were procured from eligible consented donors through the National Disease Research Interchange under protocols RGIJ1 01 001A and 002A. The biological experiments reported here were in vitro studies using human ASCs and dermal fibroblasts.

## 5. Conclusions

Taken together, these findings support a hybrid biomaterial strategy in which the structural and mechanical properties of CNF or TOCNF are combined with the biological complexity of human-derived hydrogels. CNF-human ECM and TOCNF-human ECM hybrid scaffolds gelled under physiological-temperature conditions and displayed formulation-dependent rheological properties and porous microstructures. Selected formulations supported cell-associated metabolic activity in dermal fibroblast and ASCs, qualitative compatibility with adipogenic culture, and exploratory wound-gap closure. These proof-of-concept findings support further mechanistic and preclinical evaluation, not conclusions regarding clinical efficacy. Demonstration of therapeutic benefit will require quantitative multi-donor studies, comprehensive evaluation of scaffold degradation and host responses, and validation in appropriate preclinical wound-healing models.

## 6. Patents

The work presented in this manuscript led to a patent application filed by the USDA/Obatala Sciences on April 29, 2025: U.S. Patent Application No. 19/193,179, entitled “Cellulose Nanofiber-Based Hydrogels Containing Wound-Healing Adjuvants” by Prevost et al.

## Figures and Tables

**Table 1:** Composition of hydrogel/cellulose nanofiber-based scaffolds and gelation outcome after a 30 min incubation at 37 °C.

Type of Nanofiber		% Amount of Nanofiber	%ObaGel^®^-ECM A	%ObaGel^®^-ECM B	Gelation in 30 min (Y/N)
	TOCNF	100	0	0	N
#1	TOCNF	50	10	40	Y
#2	TOCNF	30	10	60	Y
#3	TOCNF	48	2	50	Y
#4	TOCNF	20	40	40	Y
	TOCNF (3:1) ^b^				Y
	CNF	100	0	0	N
#1	CNF	50	10	40	Y
#2	CNF	30	10	60	Y
#3	CNF	48	2	50	Y
#4	CNF	20	40	40	Y
	CNF (3:1) ^b^				Y

Hybrid hydrogels were prepared using a single nanocellulose suspension, either cellulose nanofibrils (CNFs) or TEMPO-oxidized cellulose nanofibrils (TOCNFs), in combination with ObaGel^®^-ECM A and ObaGel^®^-ECM B. ^b^ The 3:1 designation indicates dilution of formulation #4 TOCNF or #4 CNF with 1× PBS at a 3:1 ratio. Diluted formulation #4 was selected as the lead condition for downstream exploratory assays based on gelation, handling, rheological behavior, transparency, and preliminary cell compatibility within the tested design space.

**Table 2:** Storage and loss moduli and viscosity of hydrogel mixtures.

Mixture	Nanofibers (*w*/*v*%)	Storage Modulus (Gʹ, Pa)	Loss Modulus (Gʺ, Pa)	Viscosity (Pa·s)
TOCNF only	1.0%	220.60	43.17	0.0562
TOCNF #4	0.2%	3309.77	726.36	0.0899
TOCNF #4 (3:1)	0.15%	3212.47	602.73	N/A
CNF only	1.0%	94.23	29.24	0.0867
CNF #4	0.20%	6806.11	1976.14	0.0945
CNF #4 (3:1)	0.15%	1337.29	284.00	N/A
ObaGel^®^-ECM	0%	64,193.1	8131.07	0.0669

## Data Availability

The original contributions presented in the study are included in the article and Supplementary Materials; further inquiries can be directed to the corresponding authors.

## Supplementary Materials

The following supporting information can be downloaded at: https://www.mdpi.com/article/10.3390/ijms27219650/s1.
