# Supplementary material for: Development of Hydrogel-Cellulose Nanofiber Hybrid Scaffolds for Potential Cutaneous Wound-Healing Applications
Source: Int J Mol Sci. 2026 Oct 28;27(21):9650. doi: 10.3390/ijms27219650 (PMC13762507; doi:10.3390/ijms27219650)
Supplement: Supplementary file 1 [file ijms-27-09650-s001.zip › ijms-4552854-supplementary.pdf]

Supplementary Information

Development of Hydrogel–Cellulose Nanofiber Hybrid Scaffolds for Potential Cutaneous Wound-Healing Applications

Judson. V. Edwards [1,*], Nicolette T. Prevost [1], Haley Lassiter [2], Katie Hamel [2] , Michael W. Easson [1], Jacobs H. Jordan [1], Emma Rogers [2], Jordan Robinson [2], Trivia Frazier [2] and Cecilia G. Sanchez [2,*]

[1] United States Department of Agriculture, Agricultural Research Service, Southern Regional Research Center, New Orleans, LA; vedwards53@yahoo.com, nicolette.prevost@usda.gov, micheal.easson@usda.gov, jacobs.jordan@usda.gov
2 Obatala Sciences Inc., New Orleans, LA; katie.hamel@obatalasciences.com, emma.rogers@obatalasciences.com, jordan.robinson@obatalasciences.com, haley.lassiter@obatalasciences.com, trivia.frazier@obatalasciences.com, and cecilia.sanchez@obatalasciences.com
*Correspondence: V.E. vedwards53@yahoo.com; +1-504-286-4360; C.S. cecilia.sanchez@obatalasciences.com, +1-504-881-3953

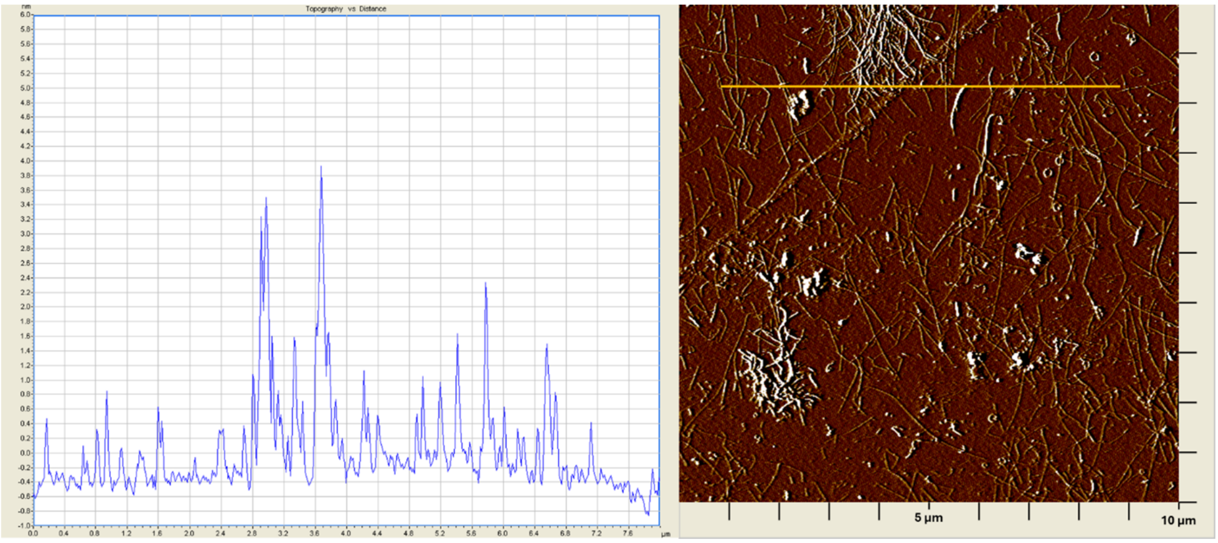

Figure S1. (A) AFM line profile of cotton-derived cellulose nanofibrils showing surface topography. (B) AFM deflection-mode image illustrating the elongated, high-aspect-ratio morphology of the nanofibrils. The fibrils exhibit lengths of approximately 1–2 µm, consistent with cellulose nanofibril structures derived from cotton. Scale bar = 5 µm.

Atomic Force Microscopy (AFM) Method

AFM images were acquired with an Agilent 500 atomic force microscope. To prepare samples for AFM, mica discs were incubated with a dilute poly-L-lysine solution (0.1 wt%) for 15 min; the discs were rinsed with deionized water to remove excess material and then blown dry under a stream of argon. One drop of CNFs suspensions (0.01 wt%) was deposited on the pretreated mica discs and air-dried for 24 h. The adhered surface was rinsed again with deionized water and dried overnight prior to data acquisition. Data was collected in contact mode using a triangular-shaped Pyrex nitride cantilever with a gold reflex coating and silicon nitride tips (0.32 N/m, 67 kHz, NanoWorld, Neuchâtel, Switzerland).

Table S1. Storage and loss moduli and viscosity of hydrogel combinations.

Formulation	Storage Modulus (G', Pa)	Loss Modulus (G", Pa)	Viscosity (Pa·s)	Nanofibers (w/v)%
TOCNF	220.60	43.17	0.0562	1.0
#1 TOCNF	4556.52	711.50	0.2054	0.5
#2 TOCNF	27573.35	5000.49	0.2375	0.3
#3 TOCNF	24101.87	6568.33	0.2077	0.48
#4 TOCNF	3309.77	726.36	0.0899	0.2
CNF	94.23	29.24	0.0867	1.0
#1 CNF	4108.58	1000.50	0.1760	0.5
#2 CNF	8647.03	1882.68	0.2158	0.3
#3 CNF	2981.86	666.77	0.1976	0.48
#4 CNF	6806.11	1976.14	0.0945	0.2
ObaGel®- ECM	64193.10	8131.07	0.0669	0.0
#4 TOCNF (3:1)	3212.47	602.73	n/a	0.15
#4 CNF (3:1)	1337.29	284.00	n/a	0.15
	**Selected at ~1 Hz**		**Selected at ~100 Hz**	%

Table S2. Image-based quantification of DAPI-positive nuclear density in dermal fibroblast cultures on human-derived biomaterials containing CNF or TOCNF.

**Condition**	**Mean DAPI+ nuclei / 10× field**	**SD**	**SEM**	**CV (%)**
**CNF**	**133.7**	**48.1**	**27.8**	**36.0**
**TOCNF**	**166.3**	**6.0**	**3.5**	**3.6**

Table S2. Nuclear density was assessed across three independent biological replicates per condition (n = 3) from the dermal-fibroblast coating experiment, after 7 days ni culture. Three 10× microscopic fields were analyzed per well and averaged within each biological replicate. Data are reported as mean nuclei per field, standard deviation (SD), standard error of the mean (SEM), and coefficient of variation (CV) from the three biological replicates.

Figure S2.

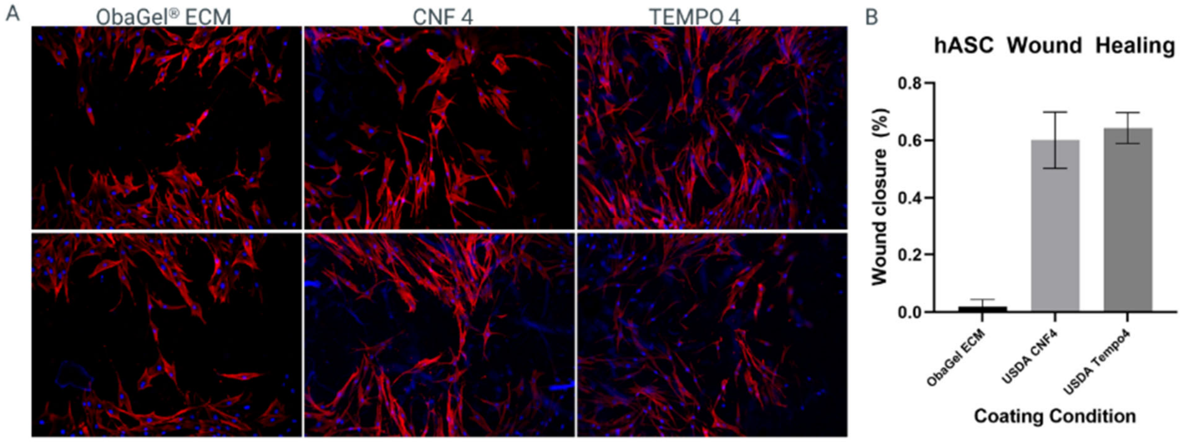

B Wound gap closure

Condition	Mean (%)	SD
ObaGel-ECM	1.9	2.4
CNF 4	64.3	5.4
TOCNF 4	60.0	9.5

C Phalloidin-positive predicted-gap area

Condition	Mean (%)	SD
ObaGel-ECM	4.2	2.2
CNF 4	9.8	0.6
TOCNF 4	8.4	5.5

Figure S2. Exploratory ASC in vitro wound-gap closure response on ObaGel®-ECM, CNF formulation #4, and TOCNF formulation #4 coatings. (A) Representative Day 2 fluorescence images from biological replicates. Hoechst 33342-stained nuclei are shown in blue, and Alexa Fluor Phalloidin 594-stained actin is shown in red. Images were acquired at 10× magnification. (B) Day 0 and Day 2 measurements from 3 biological replicates were paired within the corresponding condition and biological replicate. Wound closure was calculated as [(W0 − W2)/W0] × 100. Wound width was determined in ImageJ from three representative regions per image and reported as mean ± SD. (C) Exploratory image-based assessment of fluorescence-positive coverage within a central predicted-gap region, defined as the middle 50% of each endpoint fluorescence field. Red-positive pixels were identified using identical channel-dominance and intensity criteria across conditions. Fluorescence images were not interpreted as migration-specific endpoint.

Table S3. Qualitative wound-region observations for CNF formulation #4 and TOCNF formulation #4 compared with ObaGel®-ECM.

**Observations**	**ObaGel® ECM**	CNF formulation #4	**TOCNF**
**Residual wound/open region**	Large, clearly visible cell-free region	Much less prominent	Much less prominent
**Hoechst 33342-positive** nuclei within/near wound region	Relatively sparse	Greater presence across the field	Greater presence across the field
**Phalloidin/F-actin coverage**	Limited within the open region	Extensive	Extensive
**Cells spanning the wound region**	Limited	Clearly apparent	Clearly apparent
**Cell morphology**	Cells mainly associated with wound boundaries	Many elongated/spindle-like phalloidin+ structures	Many elongated/spindle-like phalloidin+ structures
**Apparent wound closure**	Low	Substantially greater	Substantially greater
CNF formulation #4 vs TOCNF formulation #4	—	Strong closure response	Strong closure response; broadly comparable to CNF formulation #4

Table S3. Representative Hoechst/phalloidin fluorescence images showed condition-dependent differences in wound-region coverage. CNF formulation #4 and TOCNF formulation #4 exhibited greater cellular and F-actin-positive coverage of the predicted wound region than ObaGel®-ECM, in which a prominent open region remained visible. These observations are qualitative and support, but do not independently establish, the quantitative wound-closure results shown in Figure S3B.
